# REAVER: A program for improved analysis of high‐resolution vascular network images

**DOI:** 10.1111/micc.12618

**Published:** 2020-05-15

**Authors:** Bruce A. Corliss, Richard W. Doty, Corbin Mathews, Paul A. Yates, Tingting Zhang, Shayn M. Peirce

**Affiliations:** ^1^ Department of Biomedical Engineering University of Virginia Charlottesville Virginia; ^2^ Department of Ophthalmology University of Virginia School of Medicine Charlottesville Virginia; ^3^ Department of Statistics University of Virginia Charlottesville Virginia

**Keywords:** blood vessel, image analysis, image quantification, microvascular networks

## Abstract

Alterations in vascular networks, including angiogenesis and capillary regression, play key roles in disease, wound healing, and development. The spatial structures of blood vessels can be captured through imaging, but effective characterization of network architecture requires both metrics for quantification and software to carry out the analysis in a high‐throughput and unbiased fashion. We present Rapid Editable Analysis of Vessel Elements Routine (REAVER), an open‐source tool that researchers can use to analyze high‐resolution 2D fluorescent images of blood vessel networks, and assess its performance compared to alternative image analysis programs. Using a dataset of manually analyzed images from a variety of murine tissues as a ground‐truth, REAVER exhibited high accuracy and precision for all vessel architecture metrics quantified, including vessel length density, vessel area fraction, mean vessel diameter, and branchpoint count, along with the highest pixel‐by‐pixel accuracy for the segmentation of the blood vessel network. In instances where REAVER's automated segmentation is inaccurate, we show that combining manual curation with automated analysis improves the accuracy of vessel architecture metrics. REAVER can be used to quantify differences in blood vessel architectures, making it useful in experiments designed to evaluate the effects of different external perturbations (eg, drugs or disease states).

**Abbreviations**
FN: false negatives
FP: false positives
RAVE: Rapid Analysis of Vessel Elements
REAVER: Rapid Editable Analysis of Vessel Elements Routine
TN: true negatives
TP: true positive

## 1 INTRODUCTION

The vast networks of interconnected blood vessels found in tissues throughout the body play significant roles in oxygen transport, nutrient delivery, and inflammation. The microvasculature is a key effector system in healthy and pathological conditions: serving primary roles in maintaining tissue homeostasis^1^ as well as the pathogenesis of disease.^2^ The morphological structure of a microvessel network is closely intertwined with its biological functions, and quantitative changes in structure provide evidence of an altered physiological or pathological state. Examples include vessel diameter as an indicator of vasodilation, vasoconstriction, or arteriogenesis,^2^ as well as vascular length density as an indicator of altered levels of tissue oxygenation^3^ or tissue regeneration.^4^ Since the structural architecture of microvessel networks is closely intertwined with function, changes in microvessel architecture can, therefore, be used to assess cellular and tissue level responses to disease and treatments. Confocal imaging of intact microvascular networks labeled with fluorescent tags yields images with high signal to noise^5^ and serves as a gold standard method for visualizing the structure of microvascular networks.^6^

Several image‐processing programs have been previously developed to quantify fluorescent images of microvessel architecture in an automated manner, including Angioquant,^7^ Angiotool,^8^ and RAVE.^9^ While these programs have been used in various studies, they are estimated to have a low degree of adoption by the research community relative to the multitude of studies that have quantified microvascular architecture using a manual approach.^2^ Furthermore, the publications that introduce these tools for automation lack a common method for evaluating performance and provide nonstandard forms of metrics that make comparison between them difficult.^2^ For image segmentation, manual analysis through visual inspection remains the gold standard technique,^10^, ^11^, ^12^ defined as the method accepted to yield results closest to the true segmentation. Using manual analysis as an approximation of ground‐truth^13^ can serve as a basis to compare performance between automated analysis methods by classifying disagreement from ground‐truth as error, as done previously in other applications.^14^

**FIGURE 1:**
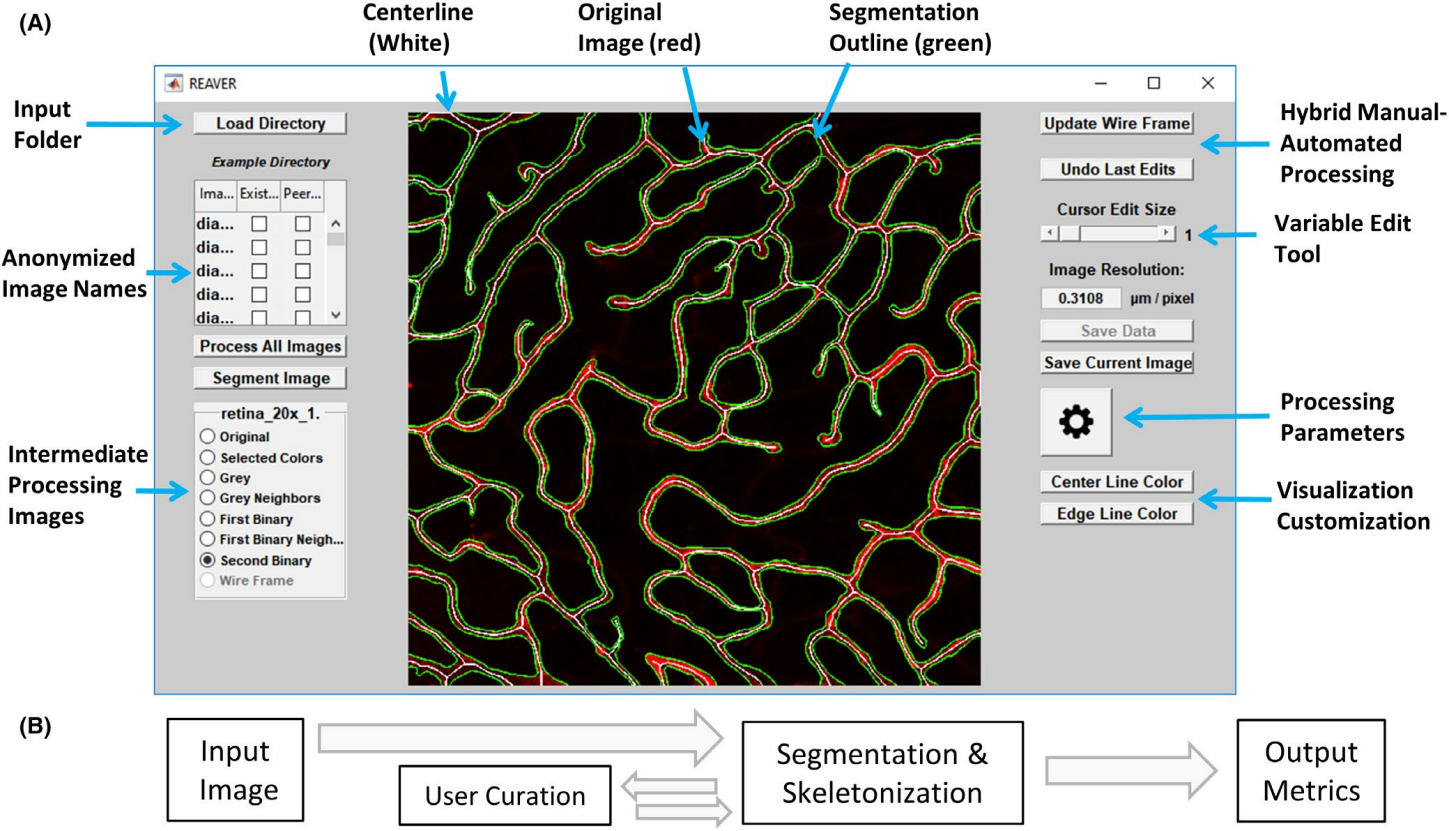
REAVER is an image analysis program for quantification of vascular networks in fluorescent images. A, Screenshot of REAVER graphical user interface. B, Flow chart of data processing pipeline

In this paper, we establish and validate a new open source tool, named REAVER, for quantifying various aspects of vessel architecture in fluorescent images of microvascular networks (Figure 1A) that uses simple image processing algorithms to automatically segment and quantify vascular networks, while offering the option for manual user curation (Figure 1B). We use a benchmark dataset of fluorescently labeled images from a variety of tissues that exhibit a broad range of vascular architectures as a means of assessing our program's general ability to automatically analyze vessel structure and minimize possibility of bias resulting from examining any single tissue. The error of REAVER's output to ground‐truth for various output metrics, including vessel length density, vessel area fraction, vessel tortuosity, and branchpoint count, is compared to the other vascular image analysis programs listed above. The accuracy of the output metrics, defined as the closeness of a measured value to ground‐truth,^15^ is measured based on absolute error.^16^, ^17^, ^18^ Precision, related to the random errors caused by statistical variability, is measured by comparing the variance of error between different programs. REAVER's effectiveness is highlighted by its greater accuracy and precision compared to all other programs. Given the ubiquity of high‐resolution fluorescent microscopy and the established need for automated, rigorous, and unbiased methods to quantify vessel architectural features, we present REAVER as an image analysis tool to further microvascular research.

## 2 MATERIALS AND METHODS

### 2.1 Code and data availability

Rapid Editable Analysis of Vessel Elements Routine source code is available under a BSD 3.0 open source license at: https://github.com/uva‐peirce‐cottler‐lab/public_REAVER. It was written MATLAB 2018a and requires the image processing toolbox to run. Benchmark image dataset and annotations are publicly available and linked from the repository main page.

### 2.2 Murine retinal harvest

All procedures were approved by the Institutional Animal Care and Use Committee at the University of Virginia and completed in accordance with our approved protocol under these guidelines and regulations. We used C57Bl6/J mice from The Jackson Laboratory (JAX stock #000664). Mice were sacrificed via CO2 asphyxiation with cervical dislocation for secondary sacrifice, eyes enucleated, and incubated in 4% PFA for 10 minutes. A single mouse was used for the 36‐image dataset across tissues (Figures 3 and 4, Figure S1) and for the retinal location dataset (Figure S3) to examine vascular heterogeneity within a single biological sample.

### 2.3 Immunohistochemistry and confocal imaging of retinas

Retinas were labeled with IB4 Lectin Alexa Fluor 647 (ThermoFisher Scientific I32450) and imaged at using a 20x objective (530 um field of view) and a 60× objective (212 μm field of view) with a Nikon TE‐2000E point scanning confocal microscope. A total of 36 2D images were obtained from z‐stacks through maximum intensity projection from six different tissues and used as a benchmark dataset for segmenting vessels and quantifying metrics of vessel architecture. To establish ground‐truth, all images were manually analyzed in ImageJ.^19^

### 2.4 REAVER algorithm

Rapid Editable Analysis of Vessel Elements Routine's algorithm was implemented in MATLAB and designed to process the image in two separate stages: a) segmentation based on intensity over local background and b) skeletonization and refinement. Segmented vasculature is identified through a combination of filtering, thresholding, and binary morphological operations. The image is first blurred with a light blurring averaging filter with an 8‐pixel neighborhood, and then, an image of the background (low frequency features) is calculated with a larger user‐defined heavier averaging filter (default: 128 pixels in length, yielding 40 µm for 20× images and 27 µm for the 60× images). To create a background‐subtracted image, the heavily blurred background image is subtracted from the lightly blurred image. The background‐subtracted image is thresholded by a user‐defined scalar (default: 0.045) to generate an initial segmentation. Next, the segmentation border is smoothed and extraneous pixels are removed with an 8‐neighborhood convolution filter that is thresholded such that only pixels with at least 4 neighbors are kept. Leveraging the domain‐specific knowledge that vessel networks are comprised of large connected components, those with area less than a user‐defined value are removed (default: 1600 pixels, yielding 155 µm^2^ for 20× and 69 µm^2^ for 60×). To further smooth segmentation borders, the complement of the segmented image is convolved with an 11‐square averaging filter (length of 3.4 µm^2^ for 20× and 2.3 µm^2^ for 60×), and values are thresholded above 0.5. To fill in holes within segmented vasculature, connected components of the complemented segmentation with less than 800 pixels (area of 77 µm^2^ for 20×, 34 µm^2^ for 60×) are set to true. The images are then thinned to compensate for a net dilation of segmentation from earlier processing steps. Finally, connected components of size less than a user‐set value (default: 1600 pixels, yielding 155 µm^2^ for 20× and 69 µm^2^ for 60×) are removed again to generate the final segmented image.

To generate the vessel centerline, the segmented image border is further smoothed with eight iterative applications of a 3‐pixel square true convolution kernel thresholded such that pixels with at least 4 neighbors are set to true. To fillsegmentation based on intensity over local in small holes and further clean the segmentation edge, the MATLAB binary morphological operations “bridge” and “fill” are applied in that order four times, along with an application of a 3‐pixel square majority filter where every pixel needs 5 or more true pixels in the square to pass. Connected components in the complement of the segmentation with pixel area less than 80 pixels (area of 7.7 µm^2^ for 20× and 3.4 µm^2^ for 60×) are set to true in order to fill in holes within segmented vessels. The initial vessel centerline is identified by applying the binary morphological “thin” an infinite number of times to the segmentation with replication padding applied; otherwise, thinned centerlines would not extend to the end of the image.

To filter out centerlines for segments that are too thin, a Euclidean distance transform is calculated from the complement of the segmented image and sampled at the pixel locations of the vessel centerline, resulting in a thickness centerline image where the vessel centerline contains values for the radius of the vessel in that region. The thickness centerline is divided into individual vessel segments via its branchpoints, the average radius calculated for each, and segments that fall below the user‐defined thickness threshold were removed (default: 3 pixels, yielding 0.9 µm for 20× and 0.6 µm for 60×). The refined vessel centerline was further cleaned with MATLAB’s “spur” and “clean” morphological operations, along with a final morphological thinning. Branchpoints and endpoints are identified with MATLAB's built in morphological operations, ignoring features located at the image border because edge effects cause false positives.

We note that while this algorithm was tested with a benchmark image dataset that included a practical range of resolutions with the default image processing set of parameters, the parameters are resolution‐dependent to some degree. We argue that the resolution range we used, images acquired at 20× and 60× magnification, represents the most relevant range of modalities for probing complete microvascular structures. Lower magnification below 20× lacked sufficient resolution to discern the structure of the smallest vessels of the microvascular network, while higher magnification over 60× sampled such small areas of vasculature that estimates of various metrics of vascular structure would be unreliable. Using resolutions far outside this range would require changing the default image processing parameters.

### 2.5 Manual analysis of benchmark dataset

To make the time demands for establishing ground‐truth manageable, a mixed‐manual analysis approach was used to analyze the benchmark dataset, where a simple set of ImageJ macros provided an initial guess for thresholding and segmenting blood vessels, and then, the user manually used the paintbrush to draw in changes required. The initial automated guess was used to save time, but there is a possibility that it biased the ground‐truth data to unfairly favor REAVER's results. To check whether bias in ground‐truth could alter statistical outcomes, a completely manual segmentation was compared to the mixed‐manual method in a subset of images from the benchmark dataset (N = 6 images, one from each tissue type, Figure S5A‐D). The completely manual analysis was conducted by a different user with no cross‐training between them to represent the worst‐case estimation of disagreement between the two methods. The disagreement of four output metrics (vessel length density, vessel area fraction, vessel diameter, and branchpoints) was examined via Bland‐Altman plots, and all metrics had no evidence of bias (N = 6 images, *P*‐values displayed in each chart, Figure S5E‐H, no multiple comparisons correction applied for conservative interpretation). The width of the confidence intervals of the mean was calculated based on the 6 sample images (normality approximation, Figure S5, ObsW CI_95_). Since the confidence interval is based on the standard error (and decreases by 1/√n), the confidence intervals for the entire benchmark dataset is estimated based on increasing the sample size from 6 to 36 images with sample standard deviation fixed (Figure S5, EstW CI_95_). We found these estimated confidence intervals were minor in size compaired to the effect sizes observed with the mean absolute error of the automated segmentation between the programs tested (Figure S5, columns labeled AngioQuant ‐ REAVER).

The mixed manual analysis used for ground‐truth for the benchmark dataset was acquired through manual curation of an initial automated threshold using macros in ImageJ to provide an initial guess of what structures in the image were considered vessels. Each image was loaded into ImageJ and an initial segmentation was calculated as a basis for manual curation. The image was segmented using a macro that removed high frequency features, applied local thresholding using the Phansalkar method,^20^ decreased noise with the despeckle function, removed binary objects of pixel area less than 100 pixels, morphologically opened the image (erosion followed by dilation), applied a median filter on the adjacent four pixel neighborhood, and finally enhanced the brightness of the image for visibility.

Following this initial segmentation, trained editors used the paintbrush tool to correct errors in the segmentation. The total time to correct the segmentation was recorded. After the segmentation was adjusted to satisfaction, another ImageJ macro was run to generate a preliminary skeleton of the image. This script applied a median filter of radius 9 and the ImageJ Skeletonize operation. Once again, the curator used the paintbrush tool to correct the automatically generated skeleton. Special care was taken to ensure the skeleton had a width of only one pixel. The total time to correct the skeleton was recorded. The segmentation was run through the same analysis code that the other automated methods were analyzed with. The curator then tagged each branchpoint in the skeleton and recorded the total count and locations. These data were used as ground‐truth to compare the automated analysis of several vessel architecture image processing pipelines.

### 2.6 Image quantification of benchmark dataset

Each software package provided different collections of metrics calculated in different ways. To fairly evaluate program performance in an unbiased fashion, a collection of four metrics was selected that could be calculated from the output data supplied by each program: specifically, the segmented vasculature image and the vessel centerline image. These output images were collected from each program and then analyzed with the same code to quantify the vessel length density, vessel area fraction, mean vessel diameter, and number of branchpoints. If these output images were not available in the program, we either inserted code to export them to disk, or captured them from the program graphical display. Some of the programs had adjustable settings that altered the image analysis process: Default image processing settings were used for all programs as a test of general performance with quantifying vascular architecture from fluorescently labeled images.

AngioTool is an open‐source package written in JAVA. We could not successfully recompile the program to access and export the output images directly and therefore had to use indirect means to obtain output images. An image was imported and processed with default settings (vessel diameter: 20, vessel intensity: [15, 255], no removal of small particles, and no filling of holes). Images of the segmentation and centerline were derived by adjusting the display settings of the postprocessed image and exported using the built‐in Windows Print Screen function to capture images without distortion or compression. AngioQuant is written in MATLAB, and the source code was modified to output the segmented image and vessel centerline. Input images were inverted prior to importation to AngioQuant and the default batch image processing parameters (kernel size: 1, edge tubules were not removed, and prune size: 10). All other numeric values were the default values for the program. RAVE was written in MATLAB, and the source code was modified to directly export the generated segmentation and skeleton for quantification. Each image was processed individually with default settings.

Rapid Editable Analysis of Vessel Elements Routine was written in MATLAB and outputs the segmentation vessel centerline images in datafiles that are stored in the same directory as the analyzed images. All images were processed in batch mode with the default values (Averaging Filter Size: 128, Grey to Binary Threshold: 0.045 Minimum Connected Component Area: 1600, Wire Dilation Threshold: 0, and Vessel Thickness Threshold: 3). Once all images were processed and the associated mat files created by REAVER, the output images were extracted from these datafiles.

Once all 36 composite images containing the segmented image and the image centerline for each program were generated, a MATLAB script was used to calculate the values for the metrics from the composites. The vessel area fraction was calculated as the fraction of true pixels in the segmentation image. Vessel length density was calculated first by obtaining vessel pixel length through summing up all pixels in the vessel centerline images, converting this to millimeter units using the image resolution, and then dividing this by the image field of view in units of mm^2^. To calculate mean vessel diameter, a Euclidean distance transform of the segmentation channel was calculated where each pixel's value was equal to its distance from the nearest false or un‐segmented pixel. Then, the skeleton channel was used as a mask to sample the distance values corresponding to the vessel centerlines to obtain the radius of vessel segments. These values were multiplied by two and subtracted by 1 to get diameter values and were converted to micrometer lengths using the image resolution. The MATLAB binary branchpoints morphological operation was used to find the branchpoints, and the number of branchpoints was calculated.

For analyzing the performance of the segmentation, true positives (TP) values for the image segmentation were calculated by taking the sum of the number of pixels that the program marked as true in the automated segmentation as well as the manual segmentation. Additionally, true negatives (TN), false positives (FP), and false negatives (FN) were calculated and used to measure segmentation accuracy, sensitivity, and specificity (see Methods: Program Evaluation Metrics).

### 2.7 Image processing execution time

The processing times for the manual data were recorded using a stopwatch while the curator was editing the segmentation and skeleton images in ImageJ. The processing times for AngioQuant, RAVE, and REAVER were all collected by adding tic/toc statements that log execution time into their MATLAB codes immediately before processing began and immediately after processing finished. This generated measurements for each program which were recorded.

Since AngioTool was provided as an executable file and the source code could not be successfully compiled without editing the code for dependency issues, reorganizing the file structure, and downloading external required libraries, the processing times were collected differently than the other three programs. The third‐party application “Auto Screen Capture” (https://sourceforge.net/p/autoscreen/wiki/Home/) was used to capture images of the AngioTool application's progress bar approximately every 15ms starting from before the start of processing to after it finished. The screenshots were automatically named as the exact time they were taken at the resolution of 1 ms The collection of screenshots was inspected to identify the start time for processing based on the mean time of the final screenshot before the progress bar changed and the one immediately after. The end time for processing was determined by taking the mean time between the final image before the progress bar completed and the image immediately after. The difference between these two mean times was taken to get a total processing time. The total measurement error from collecting processing times in this way works out to be <3% of the total processing time.

All processing times were gathered on a computer with 32GB of DDR4‐2666 RAM with CAS Latency of 15, an Intel i7‐8700K 3.7 GHz 6‐Core Processor, and a GeForce GTX 1080 graphics card with 8GB of VRAM. No overclocking, parallel processing or GPU processing was used.

### 2.8 REAVER curation analysis

Rapid Editable Analysis of Vessel Elements Routine's code was modified to include a timer object which triggered every 20 seconds to save data to disk in the same manner as when manually specified. This timer started as soon as the curator used REAVER's automatic segmentation and finished when the curator saved the curation results. After the automatic segmentation finished, the curator manually edited the segmentation using REAVER's GUI and periodically updated the wire frame button. The accuracy of metrics from the precuration output was compared to postcuration output with output from the manual analysis serving as ground‐truth. This process was initially conducted with default parameter values for REAVER’s image processing, but this yielded error with very small effect size across metrics, making it difficult to test for the potential benefits of manually curating automated results.

To test whether image curation could help with lower quality image analysis results, this process was repeated with extreme shifts in default parameters, leading to a highly suboptimal set of parameters that artificially created a lower quality segmentation with larger effect size for error to ground‐truth (Averaging Filter Size: 64, Grey to Binary Threshold: 0.07). Additionally, within the image segmentation algorithm, reducing the extent of background subtraction, and the smoothing filter was changed to a minimum of 6 neighbors instead a minimum of 4 to yield a true pixel.

### 2.9 Program evaluation metrics

The accuracy of the vessel structure metrics, defined as the closeness of a measured value to a ground‐truth,^15^ was examined with absolute error^16^, ^17^, ^18^ (Figure 3A,C,E,G). Let *Y_i,j_* be the value of a given vessel structure metric (vessel length density, vessel area fraction, branchpoint count, and vessel diameter) from the *i^th^* image and *j^th^* program, and *G_i,j_* be the corresponding ground‐truth value derived from manual analysis. We define error, *E_i,j_*, as the difference between a measurement and its corresponding ground‐truth and assess accuracy with the absolute error, *A_i,j_*:

(1) $$E_{\mathrm{ij}}=Y_{i,j}-G_{i,j}$$

(2) $$A_{i,j}=\left|E_{i,j}\right|$$

Measurements with low absolute error are considered highly accurate. We define precision^21^ *P_i,j_* of the *j^th^* program for *i^th^* image to be

(3) $$P_{i,j}=\left|E_{i,j}-{\tilde{E}}_{j}\right|,$$

where ${\tilde{E}}_{j}$ is the median of *E_ij_* across images, with *i* = 1,…, 36 images, using the variable transform from the Brown‐Forsythe test of variance^21^ (Figure 3B,D,F,H).

Additionally, we proposed metrics that quantify the agreement between each program's vessel segmentation and the ground‐truth (ie, manual segmentation) across the entire image including evaluating accuracy (S^A^), specificity (S^C^) and sensitivity (S^N^) (Figure 4A‐C). The definitions of these metrics depend on four quantities: true positives (TP), defined as the number of pixels correctly classified as vasculature (using the pixel classification result by the manual segmentation as the truth), true negatives (TN), the number of pixels correctly classified as background, false positives (FP), the number of pixels falsely identified as vasculature, and false negatives (FN), the number of pixels falsely identified as background. The metrics are^22^:

(4) $$S^{A}=\frac{TP+TN}{TP+TN+FP+FN}$$

(5) $$S^{N}=\frac{\mathrm{TP}}{TP+FP}$$

(6) $$S^{c}=\frac{\mathrm{TN}}{TN+FP}$$

For evaluating the effectiveness of manual user curation of automated segmentation, we compared the accuracy before and after user curation of automatically processed images for each vessel structure metric (vessel length density, vessel area fraction, branchpoint count, and vessel diameter) with REAVER (Figure 5A‐D). Let $Y_{i,r}^{B,D}$ denote the value of a given vessel structure metric before any user curation (superscript *B*) using default image processing parameters (superscript *D*) for the *i^th^* image from REAVER (with *r* denoting REAVER), and *G_i,r_* be the corresponding ground‐truth value (as defined previously). The absolute error $A_{i,r}^{B,D}$ used to evaluate accuracy would be defined as

(7) $$A_{i,r}^{B,D}=\left|Y_{i,r}^{B,D}-G_{i,r}\right|$$

Let $Y_{i,r}^{F,D}$ denote the value of a given vessel structure metric following user curation (superscript *F*) using default image processing parameters (superscript *D*) from REAVER. The absolute error *A^F,D^_i,r_* is

(8) $$A_{i,r}^{F,D}=\left|Y_{i,r}^{F,D}-G_{i,r}\right|$$

Error was also examined before and after user curation with a different set of internal image processing parameters set to substandard values (Figure 5E‐H). Let $Y_{i,r}^{B,S}$ denote the value of a given vessel structure metric before any user curation (superscript *B*) using substandard internal image processing parameters (superscript *S*) from REAVER (program index *j* set to *r*, the index for REAVER). The absolute error $A_{i,r}^{B,S}$ is

(9) $$A_{i,r}^{B,S}=\left|Y_{i,r}^{B,S}-G_{i,r}\right|$$

Let $Y_{i,r}^{F,S}$ denote the value of a given vessel structure metric following user curation (superscript *F*) using default image processing parameters (superscript *S*) from REAVER and not any other program (with *j* set to *r*, the program index for REAVER), The absolute error $A_{i,r}^{F,S}$ is

(10) $$A_{i,r}^{F,S}=\left|Y_{i,r}^{F,S}-G_{i,r}\right|$$

### 2.10 Summary of metric classes

Metrics used in this study are split into two main classes (Table 1). Vessel structure metrics are the measures that describe architectural features of a vessel network and used for biological research. Program evaluation metrics are measures of error calculated from vessel structure metrics or derived from differences between each program's image segmentation and corresponding manually segmented image. Program evaluation metrics are specifically used to compare error between programs and determine performance. To clarify the notation used for examining error before and after manual user curation, conventions are illustrated in Table 2.

**TABLE 1:** Metric classes

Metric class	Metric names
Vessel structure	Vessel length density, vessel area fraction, branchpoint count, vessel radius
Program evaluation	*A_i,j_*, *P_i,j_*, $A_{i,r}^{B,D}$ , $A_{i,r}^{F,D}$ , $A_{i,r}^{B,S}$ , $A_{i,r}^{F,S}$ , *S^A^*, *S^N^*, *S^C^*

**TABLE 2:** Metrics for examining user curation

	Parameter set	
	Default	Substandard
*User curation*		
Before	$Y_{i,r}^{B,D}$	$Y_{i,r}^{B,S}$
Following	$Y_{i,r}^{F,D}$	$Y_{i,r}^{F,S}$

### 2.11 Statistical analysis

To probe how the programs performed relative to one another, we compared the distributions of absolute error *A_i,j_* and precision *P_i,j_* for all pairs of programs via two‐sided paired t tests with Bonferroni adjusted p values^23^ (Figure 3). A program was identified as the best if its mean was lowest (or highest depending on the metric) and was significantly different from all other programs. For cases where the program with the best mean was not significantly different from all other programs, no conclusions were made. For choosing the best program, programs with vessel structure metrics exhibiting lower mean absolute error, standard deviation of error (Figure 3), and execution time (Figure 4D) were preferable, while programs with higher segmentation accuracy, specificity, and sensitivity (Figure 4A‐C) were considered better.

In addition to testing on accuracy and specificity, we tested whether each program had zero bias or equivalently, whether the mean error terms equals zero via a two‐tailed t test (Figure 3A,C,E,G, Bonferroni adjustment applied for 4 comparisons, one for each program). For illustrative purposes, the accuracy data from Figure 3 was also visualized as a series of Bland‐Altman plots^24^ (Figure S4), where the difference between a program's output metric and ground‐truth was plotted against their mean value for each image (meaning Y*_i,j_‐G_i,j_* was plotted against *(*Y*_i,j_ + G_i,j_)/2*). This analysis offers an illustration of a method commonly used in science to compare measurement methods and highlights the difficulty in interpreting results from several measurement methods, each with a collection of output variables.

The accuracy of vessel structure metrics was compared before and after user curation using multiple comparisons with Bonferroni‐adjusted p‐values. Since REAVER's automated results were extremely accurate (Figure 3) compared to the other programs, and consequently, the potential effect size for improvement from user curation was small, the analysis was conducted with default internal image processing parameters with REAVER and then repeated with a separate set of substandard parameters: comparing *A^B,D^_i,r_* to *A^F,D^_i,r_* (Figure 5A‐D) and then separately comparing *A^B,S^_i,r_* to *A^F,S^_i,r_* (Figure 5E‐H).

All of the test statistics examined may not follow a normal distribution. Nevertheless, the sample size of 36 images ensures the robustness of the paired t test to the violation of the normality assumptions because of the central limit theorem.^25^

Images of vessel architecture in the retina were analyzed across distinct spatial locations with regards to radius and depth from the optic nerve (Figure S3) and processed with default REAVER parameters. Each of the vessel structure metrics (vessel length density, vessel area fraction, branchpoint count, vessel radius, and others developed previously^2^) were compared at the six locations in the tissue (inner radial region, superficial depth; inner radial region, intermediate depth; inner radial region, deep depth; outer radial region, superficial depth; outer radial region, intermediate depth; outer radial region, deep depth) with pairwise two‐sample *t* tests using a Bonferroni correction as stated in Equation 11 (15 comparisons between 6 locations). A principle components analysis was conducted to visualize the qualitative separation of groups over dimensions that maximize separation.^26^

## 3 RESULTS

**FIGURE 2:**
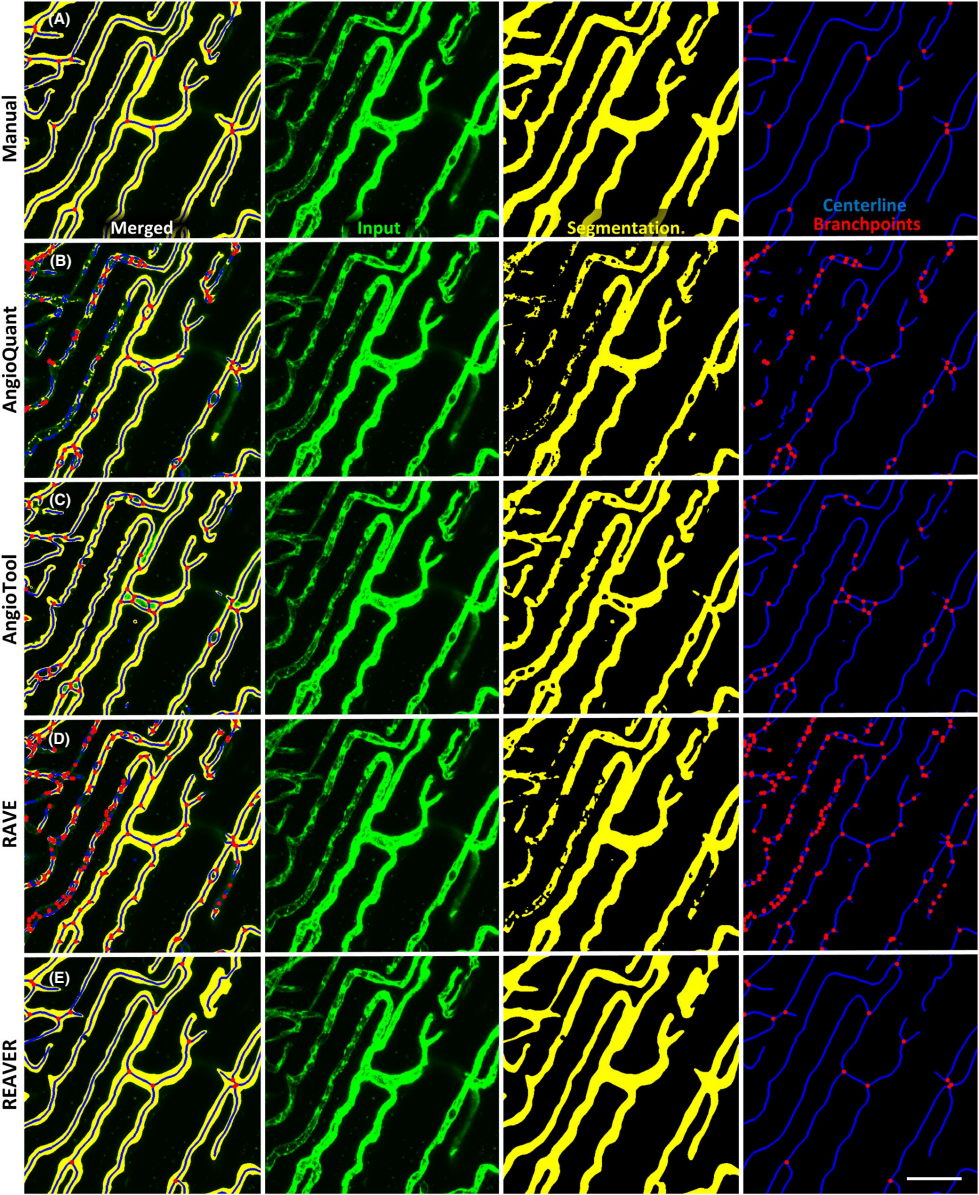
Manual segmentation and analysis can be used as ground‐truth in comparing quantification pipelines. Representative image processed with A, manual analysis, B, AngioQuant, C, AngioTool, D, RAVE, and E, REAVER with input (green), segmentation (yellow), centerline (blue), and branchpoints (red) of vascular network (scale bar 50 um)

Rapid Editable Analysis of Vessel Elements Routine was developed to analyze and quantify fluorescent images of vessel architecture using basic image‐processing techniques, including adaptive thresholding and various filters for segmentation refinement (Figure S1A‐G, see Methods: REAVER Algorithm). A dataset of images was acquired from multiple mouse tissues (Figure S2A‐F) and analyzed both manually and in an automated fashion using the REAVER, Angioquant,^7^ Angiotool,^8^ and RAVE^9^ software packages (Figure 2A‐E). The measurements quantified from the manual segmentation, along with the segmentation itself, were used as ground‐truth data. Any disagreement between the automated techniques to the ground‐truth was classified as error, allowing for comparison of performance between programs.

### 3.1 REAVER demonstrates higher accuracy and precision across metrics

**FIGURE 3:**
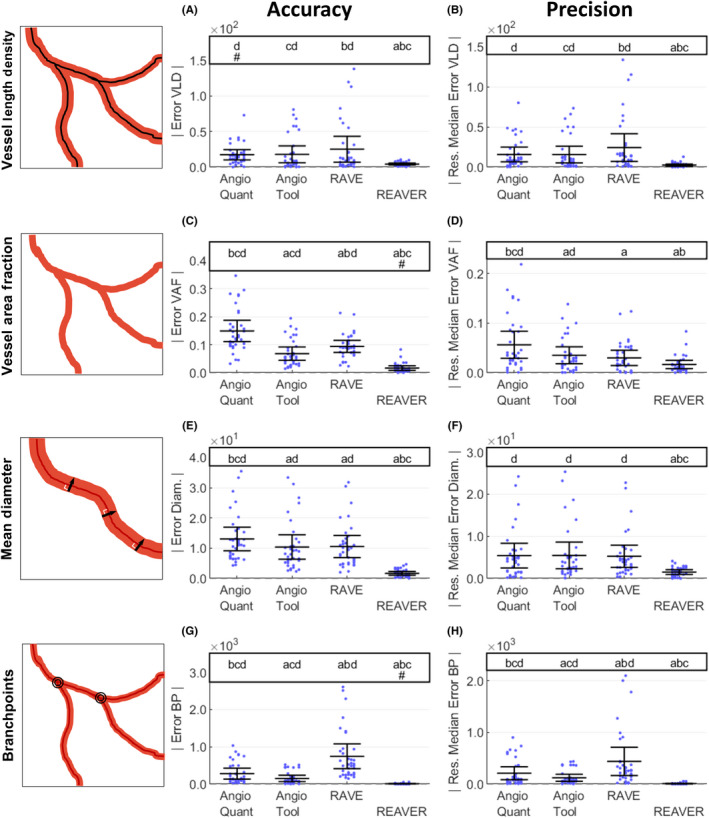
REAVER demonstrates higher accuracy and precision across metrics compared to alternative blood vessel image analysis programs. To evaluate accuracy, absolute error of A, vessel length density (mm/mm^2^), C, vessel area fraction, E, vessel diameter (µm), and G, branchpoint count compared to manual results (two‐tailed paired t tests with Bonferroni correction, 6 comparisons, α = 0.05, N = 36 images). For analysis of precision, the absolute value of residual error to group's median error for B, vessel length density (mm/mm^2^), D, vessel area fraction, F, vessel diameter (µm), and H, branchpoint count (two‐tailed paired t tests with Bonferroni correction, 6 comparisons, α = 0.05, N = 36 images). For the annotations above each plot, significant pairwise comparisons between groups with Bonferroni adjusted p‐values (letters). Groups are annotated when there is no evidence of nonzero bias with error, as determined by the origin falling within the bounds of the 95% confidence interval of the mean with Bonferroni adjustment of 4 comparisons (pound sign). Vessel metric diagrams were modified from Ref. 2

When the accuracy of vessel length density measurements was examined across the different automated image analysis tools (Figure 3A), REAVER had the lowest mean absolute error that was different from all other programs (76.5% reduction with *P* = 6.57e‐3 compared to AngioTool, the next lowest program, two‐tailed paired t tests with Bonferroni adjustment). All programs except AngioQuant had evidence of a nonzero bias revealed through individual two‐tailed *t* tests for a mean of zero (*P* < .05). When the precision of vessel length density measurements was examined, REAVER had the lowest random error that was different from all other programs (84.6% reduction with *P* = 1.61e‐3 from AngioTool, the next lowest program, two‐tailed paired *t* tests with Bonferroni adjustment) (Figure 3B).

Rapid Editable Analysis of Vessel Elements Routine also had the highest accuracy in quantifying vessel area fraction and the lowest mean absolute error that was significantly different from all the other programs (75.8% reduction of the error with *P* = 6.16e‐8 from AngioTool, next lowest program, two‐tailed paired *t* tests with Bonferroni adjustment) (Figure 3C). All programs except REAVER had a nonzero bias, revealed through the associated two‐sided t tests for a mean of zero (*P* < .05). When the precision of vessel area fraction was examined, REAVER and RAVE had the lowest random error that was different from all other programs (53.3% reduction with *P* = 8.62e‐3 from AngioTool, the next lowest program after RAVE, two‐tailed paired t tests with Bonferroni adjustment) (Figure 3D).

Rapid Editable Analysis of Vessel Elements Routine had the lowest absolute error in vessel diameter that was different from all other programs (83.9% reduction with *P* = 8.29e‐7 from AngioTool, the next lowest program, two‐tailed paired *t* tests with Bonferroni adjustment) (Figure 3E). All programs, including REAVER, exhibited evidence of nonzero bias revealed through two‐tailed *t* tests of mean zero (*P* < .05) for each individual program. In terms of the precision of the vessel diameter measurement, REAVER had the lowest random error that was different from all other programs (72.3% reduction from AngioQuant, the next lowest program, with *P* = 1.66e‐3, two‐tailed paired t tests with Bonferroni adjustment) (Figure 3F).

In terms of the accuracy of the branchpoint density measurement, REAVER had the lowest mean absolute error that was different from all other programs (94.6% reduction with *P* = 4.43e‐5 from AngioTool, the next lowest program, two‐tailed paired t tests with Bonferroni adjustment) (Figure 3G). All programs except REAVER had a nonzero bias, revealed through individual two‐tailed *t* tests for a mean of zero (*P* < .05). REAVER had the lowest random error that was different from all other programs (93.2% reduction with *P* = 4.70e‐5 from AngioTool, the next lowest program by means, two‐tailed paired *t* tests with Bonferroni adjustment) (Figure 3H).

### 3.2 REAVER exhibits higher segmentation accuracy and sensitivity with faster execution time

**FIGURE 4:**
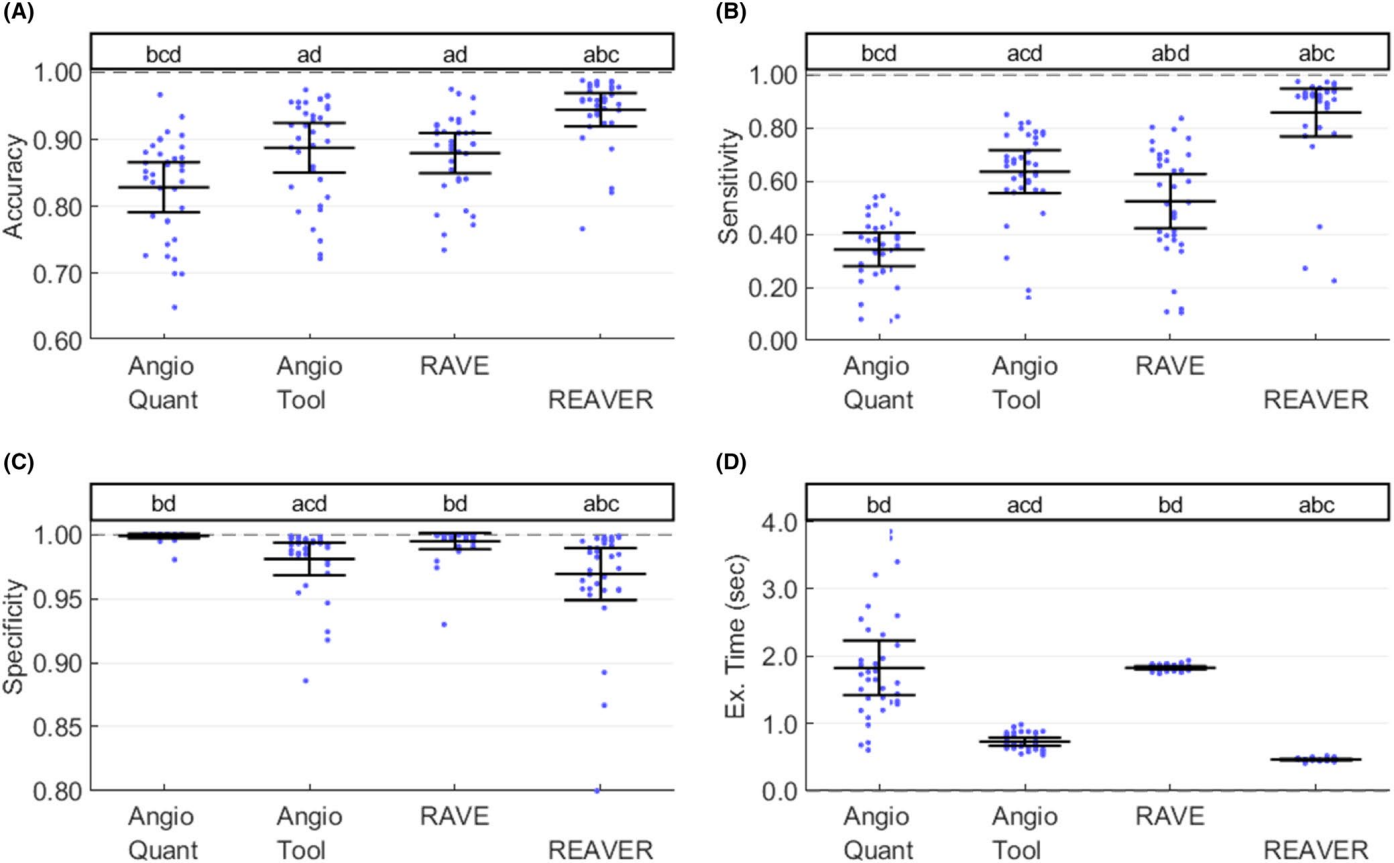
REAVER exhibits higher sensitivity and specificity with vessel segmentation, along with lower execution time compared to alternatives. Using the test dataset of images with manual analysis as ground‐truth, the (A) accuracy, (B) sensitivity, and (C) specificity of the segmentation for each program, along with (D) execution time for each image (two‐tailed paired t tests, with Bonferroni correction; 6 comparisons, α = 0.05, N = 36 images). For the annotations above each plot, significant pairwise comparisons between groups with Bonferroni adjusted p‐values below significance level (letters)

The error in the automated vessel segmentation was examined across all images in the benchmark dataset relative to the segmentation from manual analysis.^27^ REAVER had the highest mean accuracy that was different from all other programs (6.4% increase from AngioTool, the next highest program, *P* = 1.73e‐7, two‐tailed paired t tests with Bonferroni adjustment) (Figure 4A). In terms of sensitivity, REAVER had the highest mean sensitivity that was different from all other programs (34.1% increase from AngioTool, the next highest program, with *P* = 1.00e‐15, two‐tailed paired t tests with Bonferroni adjustment) (Figure 4B). In terms of specificity, RAVE and AngioQuant had higher mean specificity than the other two programs (0.4% increase from AngioTool, the next highest group, with *P* = 4.39e‐2, two‐tailed paired *t* tests with Bonferroni adjustment) (Figure 4C). With regard to execution time, REAVER had the fastest mean execution time that was different from all other programs (36.4% reduction from AngioTool, the next lowest program, with *P* = 1.8e‐16, two‐tailed paired t tests with Bonferroni adjustment) (Figure 4D). All automated program execution times were <1% of the time required for manual analysis (3089 ± 1355 seconds per image, not displayed due to orders of magnitude difference), highlighting a major benefit of automated techniques.

### 3.3 Blinded manual segmentation curation can improve accuracy of metrics

**FIGURE 5:**
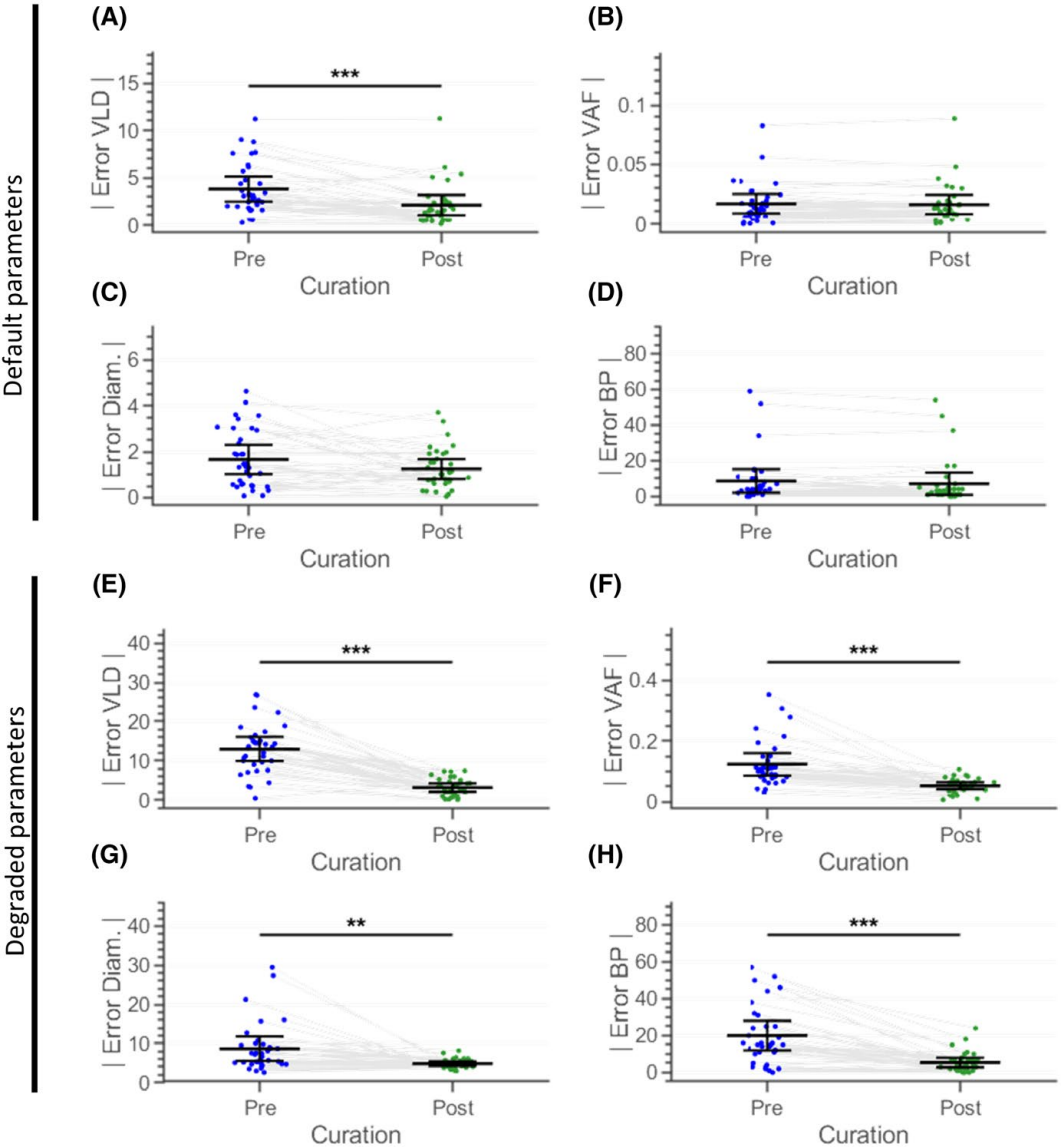
Curation of automatic image segmentation can enhance accuracy of output metrics. Comparison of error with A, vessel length density (mm/mm^2^), C, vessel area fraction, E, vessel diameter (µm), and D, branchpoint count from automated analysis using default parameters before and after manual curation of image segmentation. Comparison of error with E, vessel length density, F, vessel area fraction, G, vessel diameter, and H, branchpoint count from automated analysis using degraded parameters before and after manual curation of image segmentation (for each of the two datasets, two‐tailed paired t tests with Bonferroni correction, 4 comparisons, α = 0.05, N = 36 images)

The errors for each of the output metrics relative to the manual analysis were compared for: (a) metrics obtained by REAVER using purely automated analysis, and (b) metrics obtained by using a combination of automation paired with manual curation of the image segmentation. Using the same images and internal image processing parameters (as used in Figure 2), the absolute error across all images were compared before and after manual curation where the user was blinded to the group each image belonged to. The absolute error for vessel length density was reduced 45% (*P* = 6.4E‐5, paired two‐tailed t test with Bonferroni adjustment, Figure 5A), while there was no change to the vessel area fraction error (*P* = 1, paired two‐tailed *t* test with Bonferroni adjustment, Figure 5B). Absolute error in vessel diameter measurements had a decreasing trend, with a 25.0% reduction in absolute error (*P* = .188, paired two‐tailed *t* test with Bonferroni adjustment, Figure 5C), and absolute error in branchpoint density measurements experienced a similar decreasing trend with 17.7% reduction in absolute error (*P* = .112, paired two‐tailed *t *test with Bonferroni adjustment, Figure 5D).

Since REAVER demonstrated superior performance with this image dataset compared to the other programs, the error for many of the metrics was small, consequently lowering the potential effect size that manual curation may provide. To test whether manual curation is useful for lower quality results that could benefit more from manual curation, REAVER's internal image processing parameters were intentionally set to extreme values to produce a heavily flawed segmentation. Using the same dataset of images, user curation increased the accuracy for all of the metrics: the absolute error for vessel length density was reduced by 75.9% (*P* = 1.64e‐11, paired two‐tailed *t* test with Bonferroni adjustment, Figure 5E), the vessel area fraction absolute error was reduced 57.5% (*P* = 9.99e‐6, paired two‐tailed *t* test with Bonferroni adjustment, Figure 5F), vessel diameter absolute error was reduced 44.5% (*P* = 4.79e‐3, paired two‐tailed t test with Bonferroni adjustment, Figure 5G) and branchpoints absolute error was reduced by 73.2% (*P* = 1.36e‐6, paired two‐tailed *t* test with Bonferroni adjustment, Figure 5H).

### 3.4 REAVER reveals differences in microvascular architectures across spatial locations in murine retina

An effective microvascular image analysis program can separate between groups of images with known differences in microvascular architecture. The blood vessels of the murine retina are a well characterized microvascular network that exhibits extensive heterogeneity of vessel architecture depending location in the tissue,^2^ both with radial distance from optic disk and with each of the three discrete layers of vasculature beds: the deep plexus, intermediate plexus, and superficial capillary plexus.^28^ With a dataset of images separated by two radial distances from the center of the retina, at each of the three vascular layers (Figure S3A,B), REAVER could discern unique vessel architectural features across the metrics quantified (Figure S3D‐L). These metrics were able to achieve a partial linear separation between retina locations with the first two components of a principle components analysis (Figure S3C).

## 4 DISCUSSION

We present a novel software package, REAVER, for quantifying metrics that have been classically used to characterize microvascular network architectures. REAVER was the top performer compared to the other programs that were evaluated in terms of accuracy and precision across all four metrics of vessel architecture examined from the benchmark image dataset. We believe this is explained by the fact that previous programs were originally developed when basic image processing algorithms were computationally expensive,^8^, ^9^ or for other image modalities (such as vascular images from transmission light microscopy^7^). To minimize the possibility of biased testing, where the program we developed had an unfair advantage with our dataset, REAVER was developed using a separate dataset of images with a different labeling technique from the dataset used to design the program.^29^ To further minimize this bias, the benchmark dataset used in this study was specially designed to include a variety of mouse tissues with very diverse structural features, so efficacy was examined across tissues instead of focusing on a single tissue type.

Rapid Editable Analysis of Vessel Elements Routine may have outperformed the other programs because of its higher degree of accuracy with automated image segmentation, yielding a segmented structure closer to truth than the other programs. REAVER's higher sensitivity in segmentation demonstrated with this dataset highlights its improved ability to correctly discern foreground pixels of vessel architecture, while its lower performance in specificity suggests that other programs are better at correctly discerning background nonvessel pixels. Taken together, we interpret these results as REAVER discerning more vessel structures than other programs at the cost of generating more false positives. For this particular application, we argue that segmentation accuracy is the most important metric since vessel architecture metrics can be altered equally by false positives as well as false negatives for which pixels form blood vessels in the image segmentation. Indeed, higher sensitivity can simply be accomplished with over‐segmenting the vessel architecture, while higher specificity can be attained by under‐segmenting the image. Although REAVER had the fastest execution time compared to other programs, we argue that all programs demonstrated acceptable execution times given the low cost of computational processing power.^30^, ^31^

Performance of image analysis programs can be examined with the Bland‐Altman analysis (Figure S4A‐P), a technique that compares two measurement methods based on paired measurements^32^, ^33^ and establishes agreement if the range of the agreement interval (encompassed by the inner 95% span of the distribution of error between the two techniques) has an acceptable magnitude based on application‐specific limits defined by the researcher.^24^ This analysis is often used to compare a new measurement method with a previously developed gold standard in order to test whether the new technique can be used in place of the previous one. Although some studies assert Bland‐Altman is the only correct technique to compare methods of measurement,^34^ we highlight that it does not provide a means to compare performance of multiple measurement methods to ground‐truth. The 16 Bland‐Altman plots generated across the 4 programs and 4 metrics tested yields little insight into how well these measurement techniques performed relative to one another. Furthermore, it is frequently left up to the user to define the acceptable range for the agreement interval: in the absence of standardized approaches, this process can be influenced by perspective, opinion, and bias.

While automated results have the benefit of minimizing human interaction time required for processing images and maintaining an unbiased analysis of data, there are instances where image segmentation may perform poorly and a higher degree of accuracy is needed. To accomplish this, we propose that manual curation of segmentations derived from automated analysis, with the user blinded to group assignment, would reduce error of output metrics. While manual curation of images using default image processing parameters for REAVER showed little improvement in accuracy across all metrics, the potential effect size for improvement was small due to REAVER’s high level of accuracy and precision. To probe whether manual curation can enhance quality of results, the same images were processed using extreme values for image processing parameters that led to a poor segmentation. For this case, manual curation reduced mean absolute error approximately 60% across metrics, demonstrating the utility of hybrid approaches of data analysis where automated and manual techniques can be combined to enhance data quality. Our results indicate there are cases where manual curation can range from adding little benefit in enhancing data quality to profoundly improving the accuracy and precision of results. Using a pilot study of a small dataset of images comparing both automated results and automation with curation to ground‐truth will reveal to a researcher if curation is worth the time investment for a particular application. Furthermore, manual curation of automated segmentation represents a promising technique for efficiently generating ground‐truth analysis of images that requires much less time than purely manual techniques. It is important to note that we only investigate each program's ability to automatically segment the vasculature: many of them include several manually adjustable image processing settings (although none offer the option for direct manual curation), and there is a possibility that one of the other programs would perform better than REAVER with optimal parameters. Testing performance with manual adjustments would be a complex undertaking reserved for future research, requiring not only a fair method for identifying optimal parameters for each image and program under realistic use cases, but also evaluating how effective a user can be at identifying the optimal parameters and obtaining the optimal segmentation.

While our comparison of the precision and accuracy of four different automated image analysis programs was achieved by performing a separate comparison for each metric, in the future, it would be beneficial to compare program performance across all metrics simultaneously. This could require a method of weighting based on a metric's ability to discern alterations in a relevant biological dataset, while accounting for covariance and dependence between metrics (such as vessel length density being closely correlated with vessel area fraction for vessel networks with nearly uniform vessel diameters). The evaluation of trueness or bias, defined as the average distance between an output metric across images and ground‐truth values,^15^ is not included in this study because no method exists to statistically compare trueness between study groups since distributions must be compared to each other and their distance to zero bidirectionally at the same time. The development of such a technique would be required for discerning differences in trueness and lead to a more complete characterization of error and performance of the programs examined. Furthermore, our representation of ground‐truth could be improved by having multiple users manually analyze the images to generate a gold standard from the consensus, as done previously with image object classification.^35^

In summary, we introduce REAVER, a new software tool for analyzing architectural features in two‐dimensional images of microvascular networks, that exhibited the highest accuracy and precision for all structural metrics quantified in our study. We present REAVER as an image analysis tool to analyze high resolution fluorescence images of blood vessel networks that can be used to further microvascular research.

## 5 PERSPECTIVES

Microvascular research often requires characterizing changes in the structure of blood vessel networks, yet there is a lack of software programs to carry out these analyses. We present an open source software package, REAVER, to analyze and quantify various aspects of images fluorescent high‐resolution images of blood vessel networks. REAVER is shown to outperform other vessel architecture image analysis programs with a benchmark dataset of manually analyzed images, suggesting it as a useful tool to further microvascular research.

## CONFLICT OF INTEREST

PAY.: RetiVue, LLC (Personal Financial Interest/Employment), Genentech/Roche (Consultant). No other competing interests to disclose.

## AUTHOR CONTRIBUTIONS

BAC and RWD developed software, analyzed data, and wrote manuscript. CM generated data and aided with manuscript. TZ aided with statistical analysis and interpretation. PAY gave input on drafting of manuscript. SMP supervised the project.

## Supporting information

Figure S1‐S5

## References

[1] Davis GE , Norden PR , Bowers SLK . Molecular control of capillary morphogenesis and maturation by recognition and remodeling of the extracellular matrix: functional roles of endothelial cells and pericytes in health and disease. Connect Tissue Res. 2015;56(5):392‐402. 26305158 10.3109/03008207.2015.1066781 PMC4765926

[2] Corliss BA , Mathews C , Doty R , Rhode G , Peirce SM . Methods to label, image, and analyze the complex structural architectures of microvascular networks. Microcirculation. 2018;26:e12520. 10.1111/micc.12520 PMC6561846 30548558

[3] Pasarica M , Sereda OR , Redman LM , et al. Reduced adipose tissue oxygenation in human obesity: evidence for rarefaction, macrophage chemotaxis, and inflammation without an angiogenic response. Diabetes. 2009;58(3):718‐725. 19074987 10.2337/db08-1098 PMC2646071

[4] Tonnesen MG , Feng X , Clark RAF . Angiogenesis in Wound Healing. Invest Dermatol Symp Proc. 2000;5(1):40‐46. 10.1046/j.1087-0024.2000.00014.x 11147674

[5] Dunst S , Tomancak P . Imaging flies by fluorescence microscopy: principles, technologies, and applications. Genetics. 2019;211(1):15‐34. 30626639 10.1534/genetics.118.300227 PMC6325693

[6] Schermelleh L , Heintzmann R , Leonhardt H . A guide to super‐resolution fluorescence microscopy. J Cell Biol. 2010;190(2):165‐175. 20643879 10.1083/jcb.201002018 PMC2918923

[7] Niemisto A , Dunmire V , Yli‐Harja O , Zhang W , Shmulevich I . Robust quantification of in vitro angiogenesis through image analysis. IEEE Trans Med Imaging. 2005;24(4):549‐553. 15822812 10.1109/tmi.2004.837339

[8] Zudaire E , Gambardella L , Kurcz C , Vermeren S . A computational tool for quantitative analysis of vascular networks. PLoS ONE. 2011;6(11):e27385. 22110636 10.1371/journal.pone.0027385 PMC3217985

[9] Seaman ME , Peirce SM , Kelly K . Rapid Analysis of Vessel Elements (RAVE): a tool for studying physiologic, pathologic and tumor angiogenesis Reitsma PH, editor. PLoS ONE. 2011;6(6):e20807. 21694777 10.1371/journal.pone.0020807 PMC3111429

[10] Popovic N , Radunovic M , Badnjar J , Popovic T . Manually segmented vascular networks from images of retina with proliferative diabetic and hypertensive retinopathy. Data Brief. 2018;18:470‐473. 29900203 10.1016/j.dib.2018.03.041 PMC5996258

[11] Lebenberg J , Buvat I , Garreau M , Casta C , Constantinidès C , Cousty J , Cochet A , Jehan‐Besson S , Tilmant C , Lefort M , et al.Comparison of different segmentation approaches without using gold standard. Application to the estimation of the left ventricle ejection fraction from cardiac cine MRI sequences. Conference proceedings: … Annual International Conference of the IEEE Engineering in Medicine and Biology Society. IEEE Engineering in Medicine and Biology Society. Annual Conference. 2011;2011:2663‐2666. 10.1109/IEMBS.2011.6090732 PMC3958429 22254889

[12] Magliaro C , Callara AL , Vanello N , Ahluwalia A . A manual segmentation tool for three‐dimensional neuron datasets. Front Neuroinform. 2017;11:36. 28620293 10.3389/fninf.2017.00036 PMC5450622

[13] Ground‐truth data cannot do it alone. Nat Methods. 2011;8(11):885. 22148151 10.1038/nmeth.1767

[14] Nowotny B , Nowotny PJ , Strassburger K , Roden M . Precision and accuracy of blood glucose measurements using three different instruments. Diabet Med. 2012;29(2):260‐265. 21824188 10.1111/j.1464-5491.2011.03406.x

[15] Menditto A , Patriarca M , Magnusson B . Understanding the meaning of accuracy, trueness and precision. Accred Qual Assur. 2007;12(1):45‐47.

[16] Lehmann KG , Gelman JA , Weber MA , Lafrades A . Comparative accuracy of three automated techniques in the noninvasive estimation of central blood pressure in men. Am J Cardiol. 1998;81(8):1004‐1012. 9576161 10.1016/s0002-9149(98)00080-0

[17] Riccabona M , Nelson TR , Pretorius DH . Three‐dimensional ultrasound: accuracy of distance and volume measurements. Ultrasound Obstet Gynecol. 10.1046/j.1469-0705.1996.07060429.x 8807760

[18] Tkatchenko A , Scheffler M . Accurate molecular van der waals interactions from ground‐state electron density and free‐atom reference data. Phys Rev Lett. 2009;102(7):73005. 10.1103/PhysRevLett.102.073005 19257665

[19] Rasband WS . ImageJ. Bethesda, MD: US National Institutes of Health; 1997–2005. http://rsb.info.nih.gov/ij/

[20] Phansalkar N , More S , Sabale A , Joshi M . Adaptive local thresholding for detection of nuclei in diversity stained cytology images. In: 2011 International Conference on Communications and Signal Processing. Kerala, India: IEEE; 2011 p. 218‐220. http://ieeexplore.ieee.org/document/5739305/

[21] Brown MB , Forsythe AB . Robust tests for the equality of variances. J Am Stat Assoc. 1974;69(346):364‐367.

[22] Baratloo A , Hosseini M , Negida A , El Ashal G . Part 1: Simple definition and calculation of accuracy, sensitivity and specificity. Emergency. 2015;3(2):48‐49. 26495380 PMC4614595

[23] Chen S‐Y , Feng Z , Yi X . A general introduction to adjustment for multiple comparisons. J Thorac Dis. 2017;9(6):1725‐1729. 28740688 10.21037/jtd.2017.05.34 PMC5506159

[24] Giavarina D . Understanding Bland Altman analysis. Biochem Med. 2015;25(2):141‐151. 10.11613/BM.2015.015 PMC4470095 26110027

[25] Pagano M , Gauvreau K . Principles of Biostatistics, 2nd ed Pacific Grove, CA: Duxbury Press; 2000.

[26] Stetter M , Schiessl I , Otto T , et al. Principal component analysis and blind separation of sources for optical imaging of intrinsic signals. NeuroImage. 2000;11(5 Pt 1):482‐490. 10806034 10.1006/nimg.2000.0551

[27] Fenster A , Chiu B .Evaluation of Segmentation algorithms for Medical Imaging. In: 2005 IEEE Engineering in Medicine and Biology 27th Annual Conference. 2005 p. 7186‐7189. 10.1109/IEMBS.2005.1616166. 17281935

[28] Rust R , Grönnert L , Dogançay B , Schwab ME . A revised view on growth and remodeling in the retinal vasculature. Sci Rep. 2019;9(1):3263. 30824785 10.1038/s41598-019-40135-2 PMC6397250

[29] Corliss BA , Ray HC , Doty R , et al. Pericyte bridges in homeostasis and hyperglycemia: reconsidering pericyte dropout and microvascular structures. Cell Biology. 2019 http://biorxiv.org/lookup/doi/10.1101/704007

[30] Schatz MC , Langmead B , Salzberg SL . Cloud computing and the DNA data race. Nat Biotechnol. 2010;28(7):691‐693. 20622843 10.1038/nbt0710-691 PMC2904649

[31] Schmidt B . Bioinformatics: High Performance Parallel Computer Architectures. Boca Raton, FL: CRC Press; 2010.

[32] Altman DG , Bland JM . Measurement in medicine: the analysis of method comparison studies. J Royal Stat Soc D. 1983;32(3):307‐317.

[33] Martin Bland J , Altman DG . Statistical methods for assessing agreement between two methods of clinical measurement. Lancet. 1986;327(8476):307‐310. (Originally published as Volume 1, Issue 8476). 2868172

[34] Zaki R , Bulgiba A , Ismail R , Ismail NA . Statistical methods used to test for agreement of medical instruments measuring continuous variables in method comparison studies: a systematic review. PLoS ONE. 2012;7(5):e37908. 22662248 10.1371/journal.pone.0037908 PMC3360667

[35] Nowak S , Rüger S . How reliable are annotations via crowdsourcing: a study about inter‐annotator agreement for multi‐label image annotation. In: Proceedings of the international conference on Multimedia information retrieval ‐ MIR ’10. Philadelphia, PA: ACM Press; 2010 p. 557 http://portal.acm.org/citation.cfm?doid=1743384.1743478

